# Development of a pain management competency assessment for physiotherapy students: Integrating simulation and written assessments

**DOI:** 10.1080/24740527.2025.2512728

**Published:** 2025-07-08

**Authors:** Nathan Augeard, Jordan Miller, Geoff Bostick, Christina St-Onge, Yannick Tousignant-Laflamme, Anne Hudon, David Walton, Lesley Singer, Lynn Cooper, André Bussières, Aliki Thomas, Kadija Perreault, Susan Tupper, Lisa C. Carlesso, Peter Stilwell, Fatima Amari, Kevin Varette, Claire Ashton-James, Timothy H. Wideman

**Affiliations:** a School of Physical and Occupational Therapy, Faculty of Medicine and Health Sciences, McGill University, Montreal, Canada; b School of Rehabilitation Therapy and Health Services and Policy Research Institute, Queen’s University, Kingston, Canada; c Department of Physical Therapy, University of Alberta, Edmonton, Canada; d Département de médecine, Centre de pédagogie des sciences de la santé, Faculté de médecine et des sciences de la santé, Université de Sherbrooke, Sherbrooke, Canada; e Programme de physiothérapie, Université de Sherbrooke, Sherbrooke, Canada; f Programme de physiothérapie, École de Réadaptation, Faculté de Médecine, Université de Montréal, Montreal, Canada; g School of Physical Therapy, Western University, Ontario, Canada; h Quebec Pain Research Network, Chronic Pain Network, Hamilton, Ontario, Canada; i Canadian Injured Workers Alliance, Thunder Bay, Canada; j Département Chiropratique, Université du Québec à Trois-Rivières, TroisRivières, Canada; k École des sciences de la réadaptation, Faculty of Medicine, Université Laval, Quebec City, Canada; l School of Rehabilitation Sciences, University of Saskatchewan, Saskatoon, Canada; m School of Rehabilitation Science, Faculty of Health Sciences, McMaster University, Hamilton, Ontario, Canada; n Sydney Medical School, Faculty of Medicine and Health, The University of Sydney, Sydney, Australia

**Keywords:** Pain management education, physiotherapy, Simulation-based assessment, assessment development, interpersonal skills, validity

## Abstract

**Introduction:**

Chronic pain is a global challenge resulting in substantial healthcare costs. Despite its prevalence, gaps in pain management education persist across health professions education programs. Developing an assessment to evaluate student competency in pain management is essential to identify and address the potential impact of these disparities on learning outcomes. This study describes the development and initial evaluation of the Pain Education in Physiotherapy (PEP) competency assessment, aimed at assessing student level of competency in pain management across entrylevel physiotherapy (PT) programs.

**Methods:**

The assessment was developed using the DeVellis process, incorporating integrated knowledge translation principles and ongoing partner engagement. A steering group guided the creation of case-based multiple choice questions (MCQs) and simulation-based stations to assess competencies for pain management at different levels of Miller’s Pyramid. Initial evidence supporting the validity argument was gathered from PT students in their final semester of education (*n* = 146 for MCQs; *n* = 53 for simulations).

**Results:**

Twenty-eight MCQ items and three simulation-based stations were selected. The MCQ component showed moderate internal consistency (α = 0.65), and the simulation-based assessments demonstrated moderate internal consistency (α = 0.63) with good interrater reliability (ICC_2,1_ range: 0.73–0.86).

**Discussion:**

The PEP assessment incorporates case-based MCQs and simulation-based assessment stations to address critical interpersonal skills such as communication and empathy, often overlooked in traditional written assessments. This approach fills gaps in pain management education and provides a more comprehensive assessment tailored to PT needs.

**Conclusion:**

This assessment represents an important advancement in the assessment of pain management competencies. Its rigorous development process, partner engagement, and promising initial evaluation underscore its potential to identify gaps in pain education and help improve outcomes related to PT education.

## RÉSUMÉ

**Introduction:** La douleur chronique constitue un enjeu mondial engendrant des coûts importants pour les systèmes de santé. Malgré sa prévalence, des lacunes persistent dans la formation en gestion de la douleur au sein des programmes d’enseignement des professions de la santé. Le développement d’une évaluation des compétences des étudiants en gestion de la douleur est essentiel pour cerner et corriger les effets potentiels de ces disparités sur les acquis d’apprentissage. Cette étude décrit le développement et l’évaluation initiale de l’outil PEP, destiné à évaluer les compétences en gestion de la douleur dans les programmes d’entrée en physiothérapie.

**Méthodes:** L’évaluation a été développée selon le processus de DeVellis, en intégrant les principes de l’application des connaissances intégrée et une collaboration continue avec les partenaires. Un comité de pilotage a supervisé la création de questions à choix multiples (QCM) fondées sur des cas cliniques et de stations de simulation afin d’évaluer les compétences en gestion de la douleur à différents niveaux de la pyramide de Miller. Les premières données probantes en appui à l’argument de validité proviennent d’étudiants en physiothérapie (PT) en dernière session de formation (n = 146 pour les QCM ; n = 53 pour les simulations).

**Résultats:** Vingt-huit QCM et trois stations de simulation ont été retenues. La composante QCM a démontré une cohérence interne modérée (α = 0,65), tandis que les stations de simulation ont montré une cohérence interne modérée (α = 0,63) avec une bonne fidélité inter-juges (ICC2,1 : 0,73–0,86).

**Discussion:** L’évaluation PEP combine des QCM fondés sur des cas cliniques et des stations de simulation permettant d’évaluer des compétences interpersonnelles clés, telles que la communication et l’empathie, souvent négligées dans les évaluations écrites traditionnelles. Cette approche comble des lacunes dans la formation à la gestion de la douleur et fournit une appréciation plus globale des compétences, adaptée aux besoins des programmes de PT.

**Conclusion:** Cet outil constitue une avancée importante pour mesurer les compétences en gestion de la douleur. Son processus de développement rigoureux, l’engagement des partenaires et les résultats initiaux prometteurs soulignent son potentiel à identifier les lacunes en matière de formation et à contribuer à l’amélioration des résultats en éducation en physiothérapie.

## Introduction

Chronic pain is a leading cause of disability across the world, resulting in a substantial societal burden.^1–6^ Despite calls for improved pain management,^1,2^ many patients report that healthcare providers lack the competencies needed for effective care, such as empathetic communication, leading to misunderstanding, stigma, and prolonged disability.^7–12^

Health professions education is central to this issue, as it sets the foundation for clinical practice.^13^ Entry-level education, also known as prelicensure education, refers to the foundational training required before students enter professional practice and offers a critical opportunity for the development and assessment of pain management competencies.^13^ Entry level provides the first structured opportunity for consistent standards in the development of the competencies essential for high-quality pain management.^13^ However, inadequate entry-level pain management education for physiotherapists (PTs) and other health professionals has led to clinicians being inadequately prepared to support and provide quality care to people with chronic pain,^9,14–16^ highlighting the need for more consistent pain management education.^13–15,17–21^

Efforts to improve pain management education have included the development of interprofessional and profession-specific competencies.^22–26^ Recently, the Pain Education in Physiotherapy (PEP) competency profile was developed through a national consensus process involving educators, clinicians, and people with lived experience of pain.^22^ This profile outlines fifteen essential competencies that entry-level PTs should demonstrate to provide high-quality pain management.^22^ These include both technical (e.g., conducting a pain assessment or developing an individualized treatment plan) and interpersonal competencies (e.g., demonstrating empathy, active listening and advocacy).^22^ Robust assessments are needed to comprehensively assess these minimum competencies in entry-level physiotherapy (PT) education and ensure adequate competency development.^27–29^ Such assessments not only guide learning by identifying areas needing further development, but also confirms that graduates are equipped to meet these minimum competencies to provide high-quality patient care.^27,28^

Although chronic pain management is inherently interprofessional, entry-level education is delivered within distinct professional programs and must ensure that graduates meet the competencies specific to their scope of practice. Existing frameworks often emphasize shared competencies, such as communication and collaboration, but do not sufficiently capture the unique responsibilities and clinical reasoning processes required of PTs.^24,30^ These responsibilities demand profession-specific competencies that go beyond what is typically addressed in interprofessional assessments. Furthermore, accreditation standards and regulatory expectations for PT programs are profession-specific, reinforcing the need for targeted assessment tools. Developing a standardized assessment grounded in PT practice helps ensure that educational programs can evaluate whether new graduates are prepared to meet the unique challenges of pain management. This article provides an overview of the development process of the PEP competency assessment and the initial evidence gathered to support its validity argument for assessing students’ pain management competencies at the end of entry-level PT education.

## Theoretical framework

To guide the development and initial evaluation of the PEP competency assessment, Kane’s argument-based validity framework was adopted as the overarching framework.^31–33^ This framework conceptualizes validity as a structured argument supported by evidence across four domains: *scoring, generalization, extrapolation*, and *implications*.^31–33^ In the context of this study, *scoring* refers to the consistency and accuracy of the assessment’s scoring processes, ensuring that results are reliable and free of bias.^31–33^ *Generalization* examines whether performance on the assessment reliably represents a student’s overall competency across tasks, addressing the extent to which the assessment results can be generalized to the broader construct it aims to measure.^31–33^ *Extrapolation* focuses on whether the assessment outcomes accurately reflect the competencies required for effective pain management in clinical practice.^31–33^ Although *implications* – concerning how test scores influence decision making and educational outcomes – are an essential part of the validity argument, they fall outside the scope of this study, as it does not address the use of these scores within broader decision-making processes.^31–33^

In addition to Kane’s framework, Miller’s Pyramid of Clinical Competence, a model outlining five hierarchical levels of competence, further informed the development of the assessment.^34,35^ In this model, learners progress from theoretical knowledge (*Knows*) to applying knowledge (*Knows How*), demonstrating practical skills (*Shows How*), performing competently in real-world settings (*Does*), and, with the recent addition, reaching the stage of readiness to perform clinical duties independently (*Trusted*).^34,35^ The development of the PEP competency assessment focused on the *Knows How* and *Shows How* levels.^34,35^ Targeting these levels allows the assessment of how well students apply their theoretical knowledge to realistic situations and demonstrate competencies in a controlled environment. The *Does* and *Trusted* levels fall outside the immediate scope of a standardized assessment for university-based educators.

By integrating Kane’s validity framework with Miller’s Pyramid, this study establishes a robust theoretical foundation for developing and evaluating the PEP competency assessment. Kane’s framework ensures that the assessment’s design and findings are organized around a clear validity argument, and Miller’s Pyramid provides a targeted understanding of the specific levels of competence being assessed.

## Methods

### Creation of a steering group

A steering group of 12 members, including people living with pain (LC, LS), a recent PT graduate (NM), pain educators (AH, DW, GB, JM, TW, YTL), an expert in psychometry (CSO), and knowledge translation experts (AB, AT), oversaw all aspects of the development process. All members of the steering group had equal involvement in the planning and decision making related to this project.

### Integrated knowledge translation approach

This study was grounded in an integrated knowledge translation (IKT) approach.^36–38^ Building on previous work,^22^ we collaborated with end users and partners from the start to define assessment metrics, and create a user-friendly assessment adaptable to real-world application in pain management education.^36–38^ Throughout the process, people with lived experience, students, educators, and clinicians provided direct feedback on the assessment’s functionality, comprehensibility, and applicability, helping to assess its practicality and relevance.^36–38^ The active involvement of key partners throughout the development process aimed to ensure that the assessment closely aligns with essential pain management competencies, reducing the risk of construct underrepresentation.^39,40^ This collaborative process also provided critical feedback to enhance the clarity and usability of assessment items, helping to minimize construct-irrelevant factors and ultimately supporting the assessment’s potential to drive meaningful educational outcomes by ensuring alignment with the practical needs and experiences of patients, educators, and clinicians.^36–40^

### Design

The scale development and initial evaluation were done using the DeVellis process, a well-established 8-step process for assessment development.^41–43^ The study was approved by the Institutional Review Board of McGill University (A04-E23-20B).

#### Step 1 – defining the construct

The PEP competency profile served as the reference standard.^22^ Using the DeVellis process, the steering group then developed an assessment blueprint, by gaining consensus on the level of Miller’s Pyramid levels at which each competency would be assessed.^34,42^ This blueprint helped to determine what competencies should be assessed at what levels of Miller’s Pyramid (Supplementary Material 1). For example, developing a pain-related diagnosis or facilitating transitions in care were appropriate for the *Knows How* level, while communication skills or demonstrating empathy were best suited for the *Shows How* level.^34^

To ensure the PEP competency assessment covered all of the PEP competencies at the identified level of Miller’s Pyramid, the assessment was divided into two components. Based on partner input, both components were intended to be completed by a student within one hour each. The first component, a written exam featuring multiple-choice questions (MCQ), was used to assess the *Knows How* level. Each MCQ was framed with case-based vignettes as lead-ins, a stem, and four response options. The vignettes were designed to challenge students to showcase their ability to apply theoretical knowledge in nuanced scenarios (i.e., *Knows How*). The second component used simulation-based stations to assess competencies requiring a practical demonstration that was feasible online (i.e., *Shows How*). An online approach was chosen due to COVID-19 limitations on in-person assessments, enhancing both feasibility and accessibility. This online approach also facilitated the use of a shared set of actors portraying standardized patients across programs. The stations placed students in realistic clinical scenarios where they had to demonstrate competencies such as effective communication, and empathy. Simulation-based assessments are particularly valuable for evaluating interpersonal skills like communication and empathy, which are essential for effective pain management and are challenging to measure in written exams alone.^44–51^

#### Steps 2 and 3 – items generation & assessment format selection

At this stage, the process was divided into the generation of the MCQ items and the simulation-based stations. Steering group members identified key content domains and clinical scenarios for the PEP competencies. They developed 40 parameters (structured guidelines specifying the targeted competency, relevant content domain, and clinical scenario; see example in Table 1) to guide the creation of 120 MCQ items by lead university-based PT educators involved in pain management education. Each parameter was initially set to generate three items, allowing flexibility in selecting the questions that best aligned with content expertise and supported the validity argument for the assessment. These items were further refined based on performance and relevance, ensuring that the final items met both theoretical and statistical criteria for inclusion in the assessment.

**Table 1.** Example of a parameter to guide the MCQ item writing process.

Primary competency component(s) to target
Synthesize and interpret assessment findings to develop a pain-related classification
Primary content domains to target
Back pain
Nervous system hypersensitivity
Key scenario and problem to address
A new patient with back pain presents with a complex set of signs and symptoms that makes differential diagnosis of the underlying mechanisms a challenge (e.g., between nociceptive pain and neuropathic pain) yet indicate that pain is being primarily driven by nervous system hypersensitivity. The goal of the question is to identify that this patient’s pain is driven by nervous system hypersensitivity.

The steering group developed simulation-based stations using clinical scenarios, created from the authentic experiences of the people living with pain on our steering committee, to elicit the demonstration of the targeted competencies. To fit within the usual time and resource limitations in PT programs – typically about one hour without requiring special equipment or complex setups – the final version of the assessment was designed to include three stations. To explore a diverse range of scenarios and facilitate the inclusion of the highest performing stations, we initially generated six stations. This comprehensive approach allowed for the assessment of feasibility, effectiveness, and quality, enabling the steering group to select the best performing stations for inclusion in the final assessment.

A corresponding marking rubric was developed for each station by steering group members.^52,53^ Each of the rubrics included two broad sections: one with items specific to the station (e.g., doing a comprehensive pain assessment), and one with items shared across stations (e.g., clarity of message). Each item was scored using a scale ranging from 0 to 4, with anchors specific to each item (Supplementary Material 2).

Throughout this process, the people living with pain (LC, LS) consulted with other people with lived experience and guided the discussion to ensure that the selected competencies and assessment methods resonated with the experiences and expectations of people with lived experience.

#### Steps 4 & 5 – items review & validation item

A group of university-based pain educators (*n* = 10), international experts (*n* = 3), and Canadians living with pain (*n* = 5) reviewed the MCQs, simulation stations, and rubrics for relevance, clarity, and completeness. They were asked to score the items using a 3-level scale ranging from 1 (no reservations) to 3 (major reservations) and provide suggestions for improvement. The steering group reduced the total number of items from 120 to 60 based on experts’ scores and feedback. Items were most often excluded because they assessed knowledge at a *Knows* rather than a *Knows How* level, or because the parameter they targeted (e.g., communication or therapeutic alliance) was not well suited to a written multiple-choice format. The remaining 60 MCQ items were refined according to the suggestions provided during the rating exercise, which commonly focused on improving distractor clarity, refining language to reflect current evidence and person-centered care, and debating whether a given answer could reasonably be considered the best response.

Experts were also asked to review the simulation-based stations and share their comments regarding the scope and focus of each scenario. Based on their input, the steering group removed one station, which involved responding to an overwhelmed patient. Although it was seen as a valuable teaching activity, it was considered too broad for the scope of this assessment and difficult to evaluate fairly. The remaining five stations were revised to improve clarity, ensure they fit within the planned timeframe, and strengthen authenticity. Revisions included refining the narrative elements and adjusting the focus of each station to support consistent scoring.

Aligned with the DeVellis process and the IKT approach used, additional feedback was sought through retrospective cognitive interviews.^54–56^ Cognitive interviewing is a qualitative method used to explore how respondents understand, interpret, and respond to assessment items.^54–56^ This approach was chosen to identify potential issues with clarity, terminology, and alignment with intended constructs, without influencing initial responses. Practicing PTs and final year students from three different PT programs in Canada shared their thoughts about the MCQ items (*n* = 6 students and *n* = 4 clinicians) and simulation-based stations (*n* = 4 students and *n* = 2 clinicians).^54–56^ Conducted online by a steering group member (TW), each interview lasted approximately 60 minutes, was audio-recorded, and was analyzed thematically by the steering group.^54–56^ These interviews helped identify patterns of misunderstanding, misinterpretation, and other issues affecting clarity and construct alignment. For some of the MCQ items, concerns were raised in relation to readability, item length, vague terminology (e.g., “recent” onset of pain), and challenges in choosing a single best answer among multiple plausible options. As for the simulation stations, this process was helpful to improve technical aspects of station delivery, clarity of instructions for both students and actors, and the readability of station descriptions.

#### Step 6 – Items administration

At this stage, the steering group delivered the 60 MCQ items and five simulation-based stations without including a validation scale (e.g., desirability scale).^57,58^ This decision was anticipated from the outset, considering that the topics covered are not of a “sensitive” nature and that there is no published gold standard available for result comparison.^57,58^

The delivery process was conducted in collaboration with three Canadian entry-level PT programs. We aimed to recruit at least 50 participants in their final semester of training across three programs to allow for basic item analysis and reliability testing, consistent with the use of Classical Test Theory (CTT) in exploratory studies^59,60^ and aligned with sample sizes reported in similar educational research.^61–64^ Although larger samples are typically required for advanced psychometric analyses, CTT is well suited for early-phase testing in modest samples. This study sought preliminary evidence to support the validity argument and inform future validation work. The assessment was integrated within the compulsory curriculum of each program as a learning activity that all registered students were expected to complete. Individual results were shared with students as personal feedback to support their learning and were not included into course grades. Students were provided with the option to determine the scope of data they consented to share for research purposes, allowing them to withhold data entirely, consent to the inclusion of only their MCQ results, only their simulation-based assessment data, or both. No sample size calculation was conducted given the exploratory scope of this study and its focus on initial tool development.

The MCQ items were administered using LimeSurvey (LimeSurvey GmbH, 2006), an online survey tool approved by the McGill University Institutional Review Board. The same survey tool was used to obtain electronic consent. The simulation-based assessment was delivered online using Zoom (Zoom Video Communications Inc., 2016), a videoconference platform. Each station lasted eight to ten minutes and was recorded for later review by the evaluators.

The implementation of the MCQ simply required scheduling one hour during class time for students to complete the assessment and sharing the online survey link. Scores were automatically calculated upon completion. In contrast, the simulation-based assessment required the coordination between simulated patients (i.e., trained actors), facilitators, and evaluators. To ensure consistency, the steering group organized a training program for both the actors and evaluators. The actors participated in multiple training sessions designed to help them develop a nuanced understanding of their assigned roles, including the clinical presentations, emotional expressions, and communication styles of patients with chronic pain. This process aimed to ensure that interactions were realistic and standardized across participants, reducing variability that could undermine the assessment’s argument for validity.

The evaluators also underwent comprehensive training to ensure consistency and reliability in their scoring practices. These sessions included in-depth explanations of the rubric components, specific anchors for each Likert scale score, and practical examples of responses across varying levels of performance. Evaluators engaged in calibration exercises, such as scoring prerecorded simulations and discussing discrepancies in their ratings, to align their interpretations of the rubric criteria. This training emphasized the importance of applying the rubric consistently, supporting the *generalization* component of Kane’s validity framework by reducing interrater variability.^31–33^ During the assessment, performance on each station was independently scored by two evaluators, whose ratings were later compared to identify and address any discrepancies, further enhancing interrater reliability. These strategies ensured that evaluator expertise and consistency provided evidence supporting the *generalization* validity of the assessment.^31–33^

#### Steps 7 and 8 – Items evaluation and scale length optimization

The CTT approach was used to analyze both components of the PEP assessment.^60,65^ All analyses were conducted in the Statistical Package for Social Sciences (IBM Corp. SPSS, v26.0, Armonk, NY). Ensuring alignment with the assessment blueprint was a primary focus, serving as the main theoretical criterion to comprehensively represent each targeted competency. Statistical analysis aimed to identify the best-performing item for each parameter by balancing psychometric rigor with alignment to the intended constructs, thereby supporting the validity argument. Corrected item-total correlation (cITC) was used as an indicator of item discrimination, measuring the association between each item score and the total score.

Item difficulty, calculated by the proportion of correct responses, provided additional insight, with lower proportions indicating more challenging items. We did not apply specific cutoff values for these indicators. Instead, for each parameter, we compared the two corresponding items and selected the one that demonstrated a stronger combination of cITC and item difficulty. Although other measurement properties were not examined at this stage, this comparative approach provided preliminary evidence to support the *scoring* validity and ensured that each targeted competency was represented in the final item selection.

Following the selection of final items, a reliability analysis was performed using Cronbach’s alpha to measure internal consistency. For the simulation-based stations, interrater reliability was also evaluated using the two-way random effects model of the Intraclass Correlation Coefficient for single measures (ICC_2,1_), with both single and average measures reported alongside 95% confidence intervals to capture consistency across individual and combined rater scores.^66^ To ensure the simulation component fit within the pre-established one-hour duration, only three of the five stations could be retained. We selected the three best-performing stations based on their interrater reliability (ICC_2,1_) and their contribution to the internal consistency of the simulation component. Missing or incomplete data were handled using listwise deletion.

## Results

### MCQ written assessment

The complete data of 146 students (out of 162 respondents) across three programs were included in this analysis (Table 2). Sixteen students were excluded due to incomplete MCQ responses, as only fully completed assessments were included to ensure consistency in item-level analysis. No comparisons were made between included and excluded participants. Of the 60 items administered, four questions had good item discrimination (cITC > 0.3), 20 showed fair item discrimination (cITC 0.2–0.3), 33 demonstrated low item discrimination (cITC 0–0.19), and three questions had negative item discrimination (cITC < 0).^67^ The item difficulty coefficient ranged from 0.27 to 0.97, reflecting a wide range of challenge levels across the test items.

**Table 2.** Demographic information of participants.

**Participants in the MCQ (n = 146)**	
Sex: total (percentage)	
Female	105 (72%)
Male	41 (28%)
Age: years (SD)	
	25 (3)
**Participants in the simulation-based stations (n = 53/146)**	
Sex: total (percentage)	
Female	33 (62%)
Male	20 (38%)
Age: years (SD)	
	26 (3)

SD, standard deviation.

Of the 60 MCQ items administered, 28 were retained for the final version of the assessment (one item per parameter) based on alignment with the assessment blueprint and comparative performance. Each retained item represented one of the 28 parameters included in the final blueprint. Two parameters linked to Competency 8 (advocacy) and Competency 13 (self-reflection) were excluded due to feasibility constraints identified during pilot testing, specifically difficulties in designing written items that clearly captured these competencies. This approach allowed for the inclusion of items essential to educational goals, even when they exhibited lower discrimination values. The balance between item difficulty and discrimination supports the *scoring* validity, by ensuring the assessment measured the intended constructs while maintaining an appropriate level of challenge. Following item selection, we recalculated the psychometric properties of the 28 retained items, summarized in Table 3. Cronbach’s alpha for the 28 items was 0.65, indicating moderate internal consistency within the MCQ component of the pain management competency assessment, which further supports the reliability of the scoring process.

**Table 3.** Difficulty and discrimination of the selected MCQ items.

Item	Competencies	Mean	SD	Discrimination
Item 1	3. Diagnosis and classification	0.49	0.50	0.14
Item 2	4. Individualized treatment plans	0.83	0.38	0.24
Item 3	3. Diagnosis and classification 4. Individualized treatment plans	0.45	0.50	0.18
Item 4	2. Comprehensive assessment	0.74	0.44	0.18
Item 5	5. Transitions in care	0.92	0.27	0.30
Item 6	5. Transitions in care	0.55	0.50	0.19
Item 7	2. Comprehensive assessment 5. Transitions in care 7. Collaboration with professionals	0.53	0.50	0.15
Item 8	2. Comprehensive assessment 5. Transitions in care 7. Collaboration with professionals	0.44	0.50	0.27
Item 9	3. Diagnosis and classification	0.70	0.46	0.20
Item 10	3. Diagnosis and classification	0.75	0.44	0.15
Item 11	2. Comprehensive assessment	0.76	0.43	0.18
Item 12	3. Diagnosis and classification	0.72	0.45	0.20
Item 13	3. Diagnosis and classification 4. Individualized treatment plans 6. Monitoring and adapting	0.76	0.43	0.16
Item 14	3. Diagnosis and classification	0.90	0.30	0.18
Item 15	3. Diagnosis and classification	0.85	0.36	0.21
Item 16	3. Diagnosis and classification 6. Monitoring and adapting 7. Collaboration with professionals	0.66	0.48	0.11
Item 17	12. Social justice and cultural safety	0.89	0.32	0.26
Item 18	3. Diagnosis and classification	0.65	0.48	0.25
Item 19	2. Comprehensive assessment	0.62	0.49	0.20
Item 20	2. Comprehensive assessment 4. Individualized treatment plans	0.71	0.45	0.13
Item 21	4. Individualized treatment plans 7. Collaboration with professionals	0.87	0.34	0.18
Item 22	4. Individualized treatment plans	0.76	0.43	0.15
Item 23	4. Individualized treatment plans 6. Monitoring and adapting	0.69	0.47	0.13
Item 24	4. Individualized treatment plans 6. Monitoring and adapting	0.59	0.49	0.14
Item 25	3. Diagnosis and classification	0.65	0.48	0.24
Item 26	4. Individualized treatment plans	0.21	0.41	0.25
Item 27	4. Individualized treatment plans	0.74	0.44	0.31
Item 28	4. Individualized treatment plans	0.97	0.18	0.37

SD, standard deviation.

From a *generalization* perspective, the mean total score was 19.2 (69%) out of 28 points, with a standard deviation of 3.7, indicating moderate variability in student performance. Scores ranged from 9 (32%) to 26 (93%), providing evidence that the MCQ component effectively captures a broad spectrum of competency levels within the student cohort.

### Simulation-based assessment

Data from 53 students across three programs were available for the simulation-based assessment analysis (Table 2). The difference in sample size between the MCQ and simulation-based assessments arose because 109 students opted to share only their MCQ responses rather than both MCQ and simulation data, which included a video recording of their performance on the stations. Three out of the five initial stations (Stations 3, 4, and 5) were selected for retention in the final assessment, lasting approximately 44 minutes in total. This decision was based on their alignment with the blueprint, interrater reliability, and their contribution to the overall internal consistency of the simulation-based assessment.

Interrater reliability (ICC_2,1_) for the selected stations initially ranged from 0.66–0.83 (Table 4), reflecting good agreement among evaluators. To improve consistency, the rubrics were refined by removing or clarifying ambiguous items, which increased ICCs_2,1_ to 0.73–0.86, demonstrating excellent interrater reliability.^66^ These results support the *scoring* validity of the simulation-based assessment by ensuring that raters applied the scoring criteria consistently.

**Table 4.** ICC_2,1_ scores for Stations 3, 4 and 5 with and without adjusted rubrics.

	Original Rubrics ICC_2,1_ (95% CI)	Updated Rubrics ICC_2,1_ (95% CI)	Rubric Changes
**Station 3 (Pain assessment)**			
Single Measures	0.71 (0.54–0.83)	0.75 (0.61–0.85)	Removed rubric item d
Average Measures	0.83 (0.71–0.90)	0.86 (0.75–0.92)	
**Station 4 (Pain education)**			
Single Measures	0.50 (0.26–0.68)	0.58 (0.37–0.73)	Removed rubric items d and f
Average Measures	0.66 (0.42–0.81)	0.73 (0.54–0.85)	
**Station 5 (Treatment adjustments)**			
Single Measures	0.64 (0.44–0.78)	0.64 (0.44–0.78)	No change
Average Measures	0.78 (0.61–0.88)	0.78 (0.61–0.88)	

CI: confidence interval.

ICC_2,1_: Intraclass correlation coefficient (two-way random, single measurement).

The selected stations targeted clinical pain assessment, pain education, and treatment adjustments, contributing to an overall Cronbach’s alpha of 0.63, which indicates moderate internal consistency. Although the smaller sample size (*n* = 53) limits generalizability to the broader student population, the station design ensured representation of key competencies. This supports the *generalization* validity of the simulation-based assessment as a reliable tool for evaluating students’ performance on critical pain management skills.

## Discussion

This study developed and piloted the PEP competency assessment, designed to evaluate pain management competencies in PT students in their final semester. The final assessment included 28 case-based MCQ items and 3 simulation-based stations, each aligned with a predefined competency framework. Initial evidence supported the internal consistency and interrater reliability of both components, providing early support for the scoring and generalization components of the validity argument.

The development of the assessment was guided by a systematic blueprint based on Miller’s Pyramid, mapping each competency to the *Knows How* or *Shows How* levels. This approach provided a balanced assessment of both foundational knowledge and practical application, covering essential pain management competencies across both MCQs and simulation-based stations.^34,35^ Simulation-based stations, in particular, provided unique opportunities to assess interpersonal competencies such as empathy and communication, which are challenging to measure through written exams.^44–51^ This methodology could inform the development of similar assessment frameworks across other healthcare contexts, promoting systematic evaluations that support high-quality, patient-centered care.

Kane’s framework was used to examine evidence supporting the validity argument, focusing on the *scoring, generalization*, and *extrapolation* domains.^31–33^ Evidence for *scoring* was supported by interrater reliability in the simulation-based component and internal consistency across both components.^31–33^ Evidence related to *generalization* remains preliminary. While the sample included participants from three entry-level PT programs, the design did not aim to test score stability across programs or across multiple versions of the assessment. Further research is needed to determine whether scores would be consistent across new samples. *Extrapolation* was supported conceptually through the use of realistic, codesigned scenarios grounded in clinical experience.^31–33,36–40^ People with lived experience made direct contributions to the simulation content and rubric design by prompting the team to emphasize criteria such as active listening, validation, and shared decision making.^31–33^

These insights influenced how complex relational competencies like empathy and communication were defined, operationalized, and evaluated. The *implications* domain, which addresses how scores are used to inform educational decisions, was not examined here due to the scope of this study. We recognize that a complete validity argument should include this domain, and consistent with other early validation studies that apply Kane’s framework in stages,^68–70^ we highlight this as an important direction for future work.^31^

Beyond the use of an IKT approach and a robust theoretical backing, one of the primary innovations of this study is the development of a profession-specific assessment tailored to the unique competencies PTs should demonstrate to provide high-quality pain management.^22,29,71^ Although existing tools, such as the Pain Competency Assessment Tool (PCAT), focus on interprofessional competencies,^30^ the PEP assessment is directly aligned with the scope of PT practice, providing a more direct assessment of student preparedness for clinical practice. Developing a profession-specific tool was particularly important given that PT students are typically evaluated within their own academic programs by PT educators, and that key competencies may not be fully captured in broader interprofessional tools. In this context, a discipline-specific assessment can better support both curriculum planning and the development of professional identity among future PTs.

Although assessment alone cannot dictate curriculum content, it often drives learning priorities and can influence both student development and program design.^27,28^ The PEP competency assessment offers a structured approach to assess the essential pain management competencies within PT programs. While grounded in a competency profile, it is not tied to any specific curricular model. Rather, it was intentionally designed to be flexible and applicable across diverse educational contexts, including both competency-based and more traditional curricular structures. Although some PT programs may already include interpersonal competencies in their assessments, the PEP assessment could provide a consistent approach to prioritize these skills, ensure they are reinforced in teaching practices, and highlight their importance in pain management. This data-driven approach could help educators identify areas where students may need further development and make targeted adjustments to better prepare them for the demands of clinical practice, ultimately enhancing the quality of pain management education.

Effective pain management requires not only technical skills but also the ability to establish a therapeutic alliance, communicate clearly, demonstrate empathy, and actively listen to patients.^7,12,15,44,46,51^ However, PTs, like many healthcare providers, often struggle to fully integrate these skills in practice.^9,12,16,72^ Measuring both knowledge acquisition and interpersonal competencies could help educators better prepare future healthcare providers to manage the complexities of chronic pain.^7,12,15,44,46,51^ Students who demonstrate competency in both technical and interpersonal competencies should be better equipped to provide person-centered care when entering practice, likely resulting in shared decision-making, improved trust, and better clinical outcomes. This dual focus ensures that future PTs are better prepared to address both the physical and emotional aspects of chronic pain, which should result in more effective and compassionate care.

Implementing the PEP competency assessment in academic settings may be challenging due to the resource-intensive nature of simulation-based assessments, which require coordination among actors, facilitators, and evaluators.^73–75^ These barriers could be mitigated by using the PEP assessment as a formative teaching tool. While the PEP assessment was designed as a summative evaluation to be used at the end of entry-level PT education, future research could explore its potential for formative use, including feedback to guide student development. This would require additional “fit-for-purpose” validity testing to confirm its suitability. Integrating elements of the assessment into ongoing evaluations would allow programs to distribute resource demands over time and provide regular student feedback.^76^ For example, a simulation-based station could be used as a formative exercise to facilitate discussions on enhancing interpersonal skills.^77–80^ As the assessment continues to be refined, future work could also explore how standardized tools like this one might contribute to coordinated national or international efforts to strengthen pain management education in PT, by promoting shared expectations and supporting collaborative curriculum development.

While the initial findings supporting the validity argument are encouraging, this assessment remains in the early stages, and current results should be considered preliminary. The study was limited by a modest sample size and potential selection bias given that participation in the research component was voluntary. In addition, the study was not pre-registered on an open science platform, which may limit transparency regarding protocol adherence and analytic decisions. The simulation-based component, while valuable for assessing interpersonal skills, requires further validation to confirm its applicability across diverse educational settings and student populations. Future studies should aim to replicate and extend these findings with larger and more diverse samples to enhance *generalization* validity and examine whether demonstrated improvements in student competencies, particularly interpersonal skills, translate into meaningful improvements in clinical practice. This is particularly important given ongoing concerns about the limitations of assessing complex relational behaviors through standardized assessments. By continuing to refine and build the validity argument for the PEP assessment, this assessment could become a robust, reliable, and widely applicable resource for strengthening pain management education in PT programs.

This study contributes to the growing literature on pain management education by offering one of the first profession-specific assessments designed to evaluate both technical and interpersonal pain management competencies in PT students. Although existing assessments have largely focused on interprofessional or knowledge-based frameworks, the PEP assessment fills a gap by integrating simulation and written formats to adequately assess both knowledge and skills. This work provides a foundation for future research aimed at improving the quality and consistency of pain education in entry-level PT programs.

## Conclusion

The PEP competency assessment, developed through the DeVellis process and evaluated using CTT, addresses an identified gap in pain management education by offering PT programs a structured, validated method to assess both practical and interpersonal competencies essential for high-quality pain management. The PEP assessment’s design and evaluation processes were guided by a strong validity argument based on Kane’s framework, ensuring it effectively measures the intended competencies while remaining relevant to real-world clinical needs. Involving people with lived experience, students, clinicians, and educators throughout its development further strengthened the validity argument by ensuring the assessment aligned with diverse perspectives and clinical realities. Beyond addressing an assessment gap, the PEP assessment may offer PT educators valuable insights to better prepare students for the complexities of chronic pain care. As it gains further refinement and evaluation, this assessment holds the potential to help PTs develop the competencies needed to support people living with chronic pain.

## Supplementary Material

Supplementary Material 2.docx

Supplementary Material 1_pdf.pdf

## Data Availability

To maintain the integrity of the PEP competency assessment, particularly for future use in educational settings, we have opted not to publish the complete assessment publicly. However, we are happy to collaborate with educators and researchers who are interested in utilizing the assessment or further exploring its components. For inquiries regarding access to the assessment materials, please contact the corresponding author, Dr Timothy H. Wideman.
