# Supplementary material for: Development of a pain management competency assessment for physiotherapy students: Integrating simulation and written assessments
Source: Can J Pain. 2025 Jul 8;9(1):2512728. doi: 10.1080/24740527.2025.2512728 (PMC12239774; doi:10.1080/24740527.2025.2512728)
Supplement: Supplementary Material 2.docx [file UCJP_A_2512728_SM6756.docx]

# **Example of a station and corresponding marking rubric**

**Station**

Time to prepare for the station: 2 minutes

Background information about the patient

Jamie is 44 years old and has a long history with recurrent non-specific low back pain. Jamie is frustrated that despite all of the different treatments (medication, chiropractic, steroid injections, massage) the pain still persists. Jamie consulted you in a further attempt to eliminate the pain condition.

During your first session, your initial assessment indicated that nervous system hypersensitivity (nociplastic pain) is the primary driver of Jamie’s pain. Your prognosis is that physiotherapy, or other forms of treatment, are highly unlikely to eliminate Jamie’s back pain.

During the last session, you completed pain neuroscience education with Jamie and explained how Jamie’s pain is not caused by tissue damage. You proposed to Jamie that your treatment should focus on functional goals and coping strategies to live with the pain.

Jamie, on the other hand, wants to focus treatment on eliminating the pain and doesn’t want to resume meaningful activities, such as gardening and hiking, while experiencing pain. You are now initiating your second session with Jamie, which will be held virtually over Zoom.

Instructions

During this encounter, help Jamie consider the value of focusing treatment on functional goals, despite continued pain. Collaborate with Jamie in trying to find consensus on the overarching focus for your physiotherapy treatment.

Time to complete this station: 10 minutes

**Rubric**

**2a Provides relevant evidence-based responses**

| 0 | 1 | 2 | 3 | 4 |
| --- | --- | --- | --- | --- |
| Responses are not relevant and/or not based on evidence |  | Provides relevant evidence-based responses to some questions |  | Provides relevant evidence-based responses to all questions |

Indicators of relevant best-evidence responses to SP prompts:

- “I was thinking about what you were saying last time and it doesn’t make sense. You want me to move more, but you must not understand how bad the pain actually is. I just don’t see why you wouldn’t wait until I am better.”
  - Provides rationale for why activity-based interventions are more appropriate than pain-elimination strategies (e.g. Explaining why pain-elimination strategy is unlikely to be effective; Explanation that pain is not a direct reflection of what is happening in tissues)
- “Are you saying I have no hope of getting rid of this pain? What kind of life would that be, like living like this all of the time!”
  - Accurate communication of prognosis (NB this rubric does not evaluate how the prognosis is discussed)
- Explains how quality of life can be improved without eliminating pain
- “So, what are you actually proposing here? What would your treatment actually look like? How will it help me?”
  - Proposes an activity-based (graded) approach that is tailored to patient interests and aligned with evidence
- Other explanations that are relevant and aligned with evidence

**2b Explores patient expectations and attempts to find common ground**

| 0 | 1 | 2 | 3 | 4 |
| --- | --- | --- | --- | --- |
| Does not explore patient’s expectations and does not attempt to find common ground |  | Partially explores patient’s expectations and/or makes partially adequate attempts to find common ground |  | Fully explores patient’s expectations and adequately attempts to find common ground |

Indicators: (NB: need both exploration of expectations AND working to find common ground)

- Uses strategies to ensure that patient’s expectations are understood (e.g. probing questions, restating/confirming patient’s expectations)
- Uses the patient’s activities of interest (hiking, gardening) to build engagement in activity-based treatments and quality of life goals (e.g. linking prognosis to opportunities for improving quality of life)

**2c Clarity of message that is trying to be communicated**

| 0 | 1 | 2 | 3 | 4 |
| --- | --- | --- | --- | --- |
| Message is mostly unclear and not understandable. |  | Message is somewhat clear and understandable. |  | Message is clear and understandable. |

Indicators: (NB. clarity of communication should be considered by imagining the perspective of the SP)

- Message is coherent
- The complexity of the message is appropriate for the SP
- Language is appropriate for the SP and there is a minimal use of jargon

**2d Clarity of verbal expression**

| 0 | 1 | 2 | 3 | 4 |
| --- | --- | --- | --- | --- |
| Verbal expression is mostly unclear. |  | Verbal expression is somewhat clear. |  | Verbal expression is clear. |

Indicators: (NB: Clarity of verbal expression should be evaluated in the language used for the session (i.e. French or English))

- Verbal expression is clear, in terms of fluency, diction, grammar, tone, volume, pace

**2e Conveys empathy**

| 0 | 1 | 2 | 3 | 4 |
| --- | --- | --- | --- | --- |
| Does not demonstrate empathy and/or responds inappropriately |  | Inconsistently demonstrates empathy |  | Consistently demonstrates empathy |

 Indicators of empathy:

- Acknowledges/validates suffering and/or patient concerns, while avoiding judgemental or stigmatizing language/tone/body language
- Expressions of concern appear to be authentic/genuine
- Demonstrates that patient perspective has been considered and is understood

**2f Organization and cohesiveness**

| 0 | 1 | 2 | 3 | 4 |
| --- | --- | --- | --- | --- |
| Minimal organization and cohesiveness |  | Somewhat organized and cohesive. |  | Organized and cohesive. |

 Indicators of organization and cohesiveness:

- Flexibility in adapting to patient concerns/responses
- Uses time effectively (e.g. avoids repetition)
- Logical/clear structure and flow to the encounter
- Able to integrate responses to patient questions/concerns within overarching purpose of the encounter
- Structures the session in a purposeful manner that aligns with the station objective (i.e. using a collaborative approach to trying to help the patient understand the value of functional goals and find common ground)

**2g Uses a collaborative approach**

| 0 | 1 | 2 | 3 | 4 |
| --- | --- | --- | --- | --- |
| Does not use a collaborative approach. |  | Inconsistently uses a collaborative approach |  | Consistently uses a collaborative approach. |

 Indicators of a collaborative approach:

- Uses strategies to assess patient’s understanding (e.g. asks patient if they have understood; uses a brief teach-back strategy)
- Uses strategies to encourage patient engagement
- Listens to patient’s response
- Tailors communication based on patient’s response
- Rolls with resistance

**2h Global rating**

Considering the goal of this station was to *use a person-centred and evidence-based approach to finding common ground on the focus for PT treatment*, please rate the student’s overall level of competency on this station:

| Well below entry-level | Slightly below entry-level | Meets minimal entry-level | Slightly above entry-level | Well above entry-level |
| --- | --- | --- | --- | --- |
