# Supplementary material for: Development of a pain management competency assessment for physiotherapy students: Integrating simulation and written assessments
Source: Can J Pain. 2025 Jul 8;9(1):2512728. doi: 10.1080/24740527.2025.2512728 (PMC12239774; doi:10.1080/24740527.2025.2512728)
Supplement: Supplementary Material 1_pdf.pdf [file UCJP_A_2512728_SM6753.pdf]

	Component of assessment	Simulation (shows how)			Multiple Choice (knows how)																											
	Question/station	3	4	5	1	2	3	4	5	6	7	8	9	10	11	12	13	14	15	16	17	18	19	20	21	22	23	24	25	26	27	28
	**Components**																															
**Competency 1: Develop a therapeutic alliance with people experiencing pain**		x	x	x																												
**Competency 2: Perform a comprehensive assessment with the person living with pain that uses appropriate tools and strategies to explore and evaluate the lived experience of pain, as well as the mechanisms underlying pain and the physical, psychological and socio-environmental factors that influence pain.**	Accurately screen, identify and triage conditions that require immediate action, that would benefit from non-urgent referral to other services, or that would benefit from modifications of to the pain management plan							x			x	x																				
	Accurately identifies pain-related impairments, activity limitations, participation restrictions																															
	Use appropriate tools and strategies to explore the lived experience of the person living with pain			x																												
	Use appropriate tools and strategies to evaluate the mechanisms underlying pain																															
	Uses appropriate tools and strategies to evaluate socio-economic factors associated with pain			x																												
	Uses appropriate tools and strategies to evaluate psychological factors associated with pain			x																				x								

	Use appropriate tools and strategies to evaluate physical factors associated with pain														x								x									
**Competency 3: Synthesize and interpret assessment findings to develop a pain-related diagnosis and/or classification, and to generate a prognosis.**	Synthesize and interpret assessment findings to develop a pain-related diagnosis													x					x													
	Synthesize and interpret assessment findings to develop a pain-related classification				x								x			x		x		x		x							x			
	Synthesize and interpret assessment findings to develop a pain-related prognosis						x										x	x														
**Competency 4: Develop and implement an individualized treatment plan that is based on the assessment findings and goals of the person living with pain.**	Identify goals to guide treatment plan																								x							
	Develop individualized treatment plan					x	x										x							x						x	x	x
	Implement individualized treatment plan		x																							x	x	x			x	
**Competency 5: Facilitate appropriate transitions in care for the person living with pain.**	Facilitate the transfer of care between different health professionals, settings, or services, and make the links to appropriate resources									x	x	x																				
	Facilitate discharge from care								x																							
**Competency 6: Use appropriate tools and strategies to monitor and evaluate the effectiveness of the treatment plan for the person living with pain, and adapt care accordingly.**	Monitor and evaluate treatment plan	x																														
	Adapt care accordingly	x	x														x			x							x	x				
**Competency 7: Collaborate with relevant professionals in a manner that fosters an integrated, patient-centred approach to pain management.**	Collaborate with relevant professionals										x	x								x					x							

Competency 8: Advocate with, and on behalf of, people living with pain, at the level of the individual, family and/or care providers, institution and community.	Advocate at the level of the individual																															
	Advocate at the level of the family or healthcare providers																															
	Advocate at the level of the institution																															
	Advocate at the level of the community																															
**Competency 9: Use a person-centered approach to pain management**		x	x	x																												
**Competency 10: Support and promote the autonomy of the person living with pain and foster partnership in their care.**	Promote autonomy																															
	Foster partnership	x	x	x																												
**Competency 11: Communicate with people living with pain in a way that is tailored to their individual needs and abilities, demonstrates active listening and empathy, and validates their lived experience.**	Tailoring communication to the individual and context	x	x	x																												
	Active listening	x	x	x																												
	Validation	x	x	x																												
	Empathy	x	x	x																												
**Competency 12: Make practice decisions that are informed by principles of social justice, inclusiveness and equity, and promote cultural safety.**	Social justice																															
	Inclusiveness																															
	Equity																															
	Cultural safety																				x											
Competency 13: Engage in critical self-reflection that fosters continuous professional growth and development.	Self-reflection																															
**Competency 14: Integrate best research evidence, clinical expertise and patient values when making practice decisions.**	Best research evidence	x	x																													
	Clinical expertise	x	x																													
	Patient values	x	x																													
**Competency 15: Use a safe, ethical and compassionate approach to care.**	Safe																															
	Ethical																															
	Compassionate			x																												
